# Nanostructured copper-organic frameworks for the generation of sulphate radicals: application in wastewater disinfection

**DOI:** 10.1007/s11356-023-29394-9

**Published:** 2023-09-06

**Authors:** Alba Giráldez, Antía Fdez-Sanromán, Daniel Terrón, M Angeles Sanromán, Marta Pazos

**Affiliations:** Department of Chemical Engineering, CINTECX, Universidade de Vigo, Campus As Lagoas-Marcosende, 36310 Vigo, Spain

**Keywords:** Copper, Disinfection, *Escherichia coli*, HKUST-1, Peroxymonosulphate, Sulphate radicals

## Abstract

In recent years, the presence of pathogens in the environment has become an issue of widespread concern in society. Thus, new research lines have been developed regarding the removal of pathogens and persistent pollutants in water. In this research, the efficacy of nanostructure copper-organic framework, HKUST-1, has been evaluated for its ability to eliminate *Escherichia coli* and generate sulphate radicals as catalyst for the treatment of effluents with a high microbiological load via peroxymonosulphate (PMS) activation. The disinfection process has been optimized, achieving complete elimination of *Escherichia coli* growth after 30 min of testing using a concentration of 60.5 mg/L HKUST-1 and 0.1 mM of PMS. To overcome the operational limitations of this system and facilitate its handling and reutilization in a flow disinfection process, HKUST-1 has been efficiently encapsulated on polyacrylonitrile as a novel development that could be scaled up to achieve continuous treatment.

## Introduction

In the last years, wastewater treatment plants (WWTPs) have been working on effluent regeneration, to eliminate the pollutants present in it, to convert it into effluent that can be released into the water cycle. Depending on the nature of the pollutants present in this water, different processes have been applied in WWTPs and insufficient treatment can adversely affect human health and the environment (Zhang et al. 2020). However, emerging contaminants have appeared in the water cycle, and inside this group of pollutants are chemicals and other particles present in water such as nutrients, heavy metals, or microorganisms (Miraji et al. 2016; Ahmed et al. 2021). A few illustrative examples of these emerging contaminants are pharmaceuticals (analgesics, antibiotics, anti-inflammatories, etc.), personal care products (cosmetics, sunscreen agents, etc.), hormones, dioxins, pesticides, surfactants, bacteria (*Escherichia coli* (*E. coli*), *Enterococci*, etc.), polycyclic aromatic hydrocarbons, nanomaterials, etc. (Naidu et al. 2016; Majumder et al. 2021). Since many of these pollutants are extremely stable chemical and microbial agents, therefore, traditional wastewater treatment plants are not able to remove and degrade them properly, which has forced research to improve and innovate wastewater disinfection and purification methods (Alvarino et al. 2018).

In this issue, one of the most promising innovations is the application of advanced oxidation processes (AOPs) to wastewater. This approach is characterized by high microbiological disinfection efficiency, low cost, and lower environmental impact. These processes generate highly reactive oxygen species (ROS) such as hydroxyl radical, hydrogen peroxide, ozone, singlet oxygen, sulphate radical, and superoxide radicals. These ROS degrade organic matter and achieve microbial removal (Hasani et al. 2019; Diaz Soria 2021). The in situ generation of these ROS initiates oxidation reactions in water to damage the membrane, proteins, lipids, enzymes, DNA, and RNA of pathogenic microorganisms (Kanakaraju et al. 2018).

Among the different AOPs, the generation of sulphate radical for bacterial disinfection has gained the attention of the scientific community, because this treatment can be applied in a wider pH range compared with the traditional Fenton reaction (Chen et al. 2021). For the generation of sulphate radicals, different oxidants can be used such as persulphate, peroxymonosulphate (PMS), or peroxydisulphate. In addition, the activation of the oxidants can be performed by metal transition (free or supported), light radiation, microwave, or carbonaceous materials (Oh et al. 2016). In the following equations (1), (2) and (3), the main reactions of activation of PMS are summarized:

1 $${{\mathrm{HSO}}_5}^{-}\longrightarrow {\mathrm{HO}}^{\bullet }+{\mathrm{SO}}_{4}{^{\bullet -}}$$

2 $${{\mathrm{HSO}}_5}^{-}+{\mathrm{e}}^{-}\longrightarrow {\mathrm{OH}}^{-}+{\mathrm{SO}}_{4}{^{\bullet -}}$$

3 $${{\mathrm{HSO}}_5}^{-}+\mathrm{energy}\ \mathrm{input}\longrightarrow {\mathrm{OH}}^{-}+{\mathrm{SO}}_{4}{^{\bullet -}}$$

The sulphate radicals generate a powerful disinfection action. According to Xia et al. (2018), these radicals provoke the pathogen removal as a result of the rupture of the cell envelope and the inhibition of ATP formation by restraining the enzymes located in the respiratory chain.

Recently, novel heterogeneous catalysts metal-organic frameworks (MOFs) have been used for the generation of sulphate radicals (Kohantorabi et al. 2021). These materials have proven to be a very promising class of materials because pore size, pore shape, and specific surface area can be manipulated with the choice of organic building blocks and metal clusters (Riley et al. 2020; Fdez-Sanromán et al. 2022b). Therefore, the use of these materials for the removal of organic compounds in wastewater has been a hot topic, and numerous studies have focused on this issue (Han et al. 2022). However, limited studies have evaluated their use as catalysts for disinfection processes (Hongyu et al. 2022; Fdez-Sanromán et al. 2023). Several studies have demonstrated that MOFs alone or encapsulated in different organic supports (corncobs or cellulose) can act as *E. coli* disinfecting agents. Although this disinfection has not been completed, even working at concentrations ranging from 330 to 480 mg/L and after 24 h (Table 1), achieving complete disinfection with these materials is a challenge too. In this regard, only our previous study (Fdez-Sanromán et al. 2023) has shown that the MOF CuFe-(BDC-NH_2_)_R_ 250 mg/L can be used for PMS activation for total bacteria disinfection in 60 min.

**Table 1:** Disinfection of *E. coli* by MOFs according to reported literature

MOF	Concentration (mg/L)	Disinfection efficiency (%)	References
HKUST-1	431	93^Ω^	Duan et al. (2019)
Ag/UiO-66-NH_2_	330	70	Tian et al. (2022)
ZIF-8	480	69	Duan et al. (2019)
Ag/BUC-51	400	98^Ω^	Liu et al. (2018)
HKUST-1@Cellulose	431	99	Abdelhamid and Mathew (2022)
HKUST-1@OCB	431	90^Ω^	Duan et al. (2019)
ZIF-8@Cellulose	480	70	Abdelhamid and Mathew (2022)
ZIF-8@OCB	480	45	Duan et al. (2019)
MOF-5@Cellulose	-	98^Ω^	Lu et al. (2018)

^Ω^Less than 99% cannot ensure a complete disinfection

*OCB*, TEMPO oxidized corncobs; *BUC*, coordination polymers

-Not provided

As a novelty in the present study, sulphate radical action will be evaluated to disinfect wastewater with high microbiological load, using PMS as oxidant activated with a low concentration of a nanostructure copper-organic framework (HKUST-1). The catalytic action of the heterogeneous copper-organic framework will be compared with free copper to evaluate the effectiveness of both. This is because the recent advances that have been made in nanotechnology have achieved to design MOFs that are highly reactive, have a large surface area, are non-toxic, have great stability against extreme conditions, and have easy retention after disinfection treatment (Noriega-Treviño et al. 2012; Diaz Soria 2021).

Therefore, the present research is focused on the evaluation and optimization of the different variables involved in the disinfection process, such as the treatment time, PMS concentration, and catalyst concentration (Cu (II) or HKUST-1). The assays use synthetic wastewater, with similar characteristics to effluents spiked with *E. coli* bacteria. Furthermore, to enhance the scalability and reusability of the catalyst, the encapsulation of the HKUST-1 in polyacrylonitrile (PAN) beads will be evaluated to overcome the handling problems and the performance will be assessed in a reactor working continuous flow.

## Materials and methods

### Microorganism and chemicals

*E. coli* strain NCIMB 9483 (CECT 102) from the Spanish Type Culture Collection (CECT) of the University of Valencia was selected for this study. This bacterium is considered a key microorganism indicator of water quality according to the World Health Organization because it has a direct relationship with pathogenic microorganisms that may be present in these waters and are difficult to identify and quantify (Villanueva Jaramillo 2020).

The growth medium for the disinfection test was the Meat Peptone Broth (MPB), which was composed of bacteriological peptone, 10 g/L (PanReac AppliChem); meat extract, 5 g/L (PanReac AppliChem); and sodium chloride, 5 g/L (Carlo Erba Reagents, 96.0%). For cryopreservation of the strain, the same medium was used but supplemented at 25% (v/v) with glycerol (Sigma-Aldrich, ≥99.0%) (Blanco-Canella et al. 2022).

The solid culture medium (MPB-agar) for the evaluation of the effectiveness of the disinfection test had the same composition as the MPB medium but with the addition of 20 g/L of agar (Scharlau) reagent.

The synthetic wastewater was prepared with the following components: 0.268 g/L ammonium sulphate ((NH_4_)_2_SO_4_; VWR chemicals), 0.06 g/L magnesium sulphate heptahydrate (MgSO_4_·7H_2_O; Scharlau; ≥98.0%), 0.006 g/L manganese (II) sulphate monohydrate (MnSO_4_·H_2_O; Scharlau, ≥99.0%), 0.0003 g/L iron (III) chloride hexahydrate (FeCl_3_·6H_2_O; Scharlau, 97.0%), 0.006 g/L calcium chloride dihydrate (CaCl_2_·2H_2_O; Scharlau, ≥99.0%), 1 g/L glucose (C_6_H_12_O_6_; Scharlau) (Fdez-Sanromán et al. 2022a).

In addition, for the disinfection test, it was necessary to prepare a buffered peptone water solution (15 g/L; PanReac AppliChem), a 0.9% (v/v) saline solution (NaCl; Scharlau, ≥99.0%), and a neutralizing solution, which was composed of 4% (w/v) sodium thiosulphate (Na_2_S_2_O_3_; VWR chemicals, ≥98.0%) and 0.25 M potassium phosphate monobasic (KH_2_PO_4_; VWR chemicals) and adjusting the pH to 7.0 with a 4-M sodium hydroxide solution (Sigma-Aldrich, ≥98.0%) (Raffellini et al. 2011).

The used catalysts were copper sulphate pentahydrate (CuSO_4_·5H_2_O) and HKUST-1 (C_18_H_6_Cu_3_O_12_) provided by Sigma-Aldrich. From the same supplier, the PMS (2KHSO_5_·KHSO_4_·K_2_SO_4_) was obtained, which was used in a 300-mM solution. To encapsulate HKUST-1 in beads, PAN (Sigma-Aldrich) and dimethyl sulfoxide (DMSO; MedChemExpress; 99.0%) were used (Riley et al. 2020).

For the scavenging assays, the following reagents were used: methanol (CH_3_OH; ≥99.0%), potassium iodide (KI; ≥99.0%), sodium azide (NaN_3_; ≥99.0%), and Reactive Black 5 (RB5; C_26_H_21_N_5_Na_4_O_19_S_6_; ≥50.0%), provided by the supplier Sigma-Aldrich.

All the solutions were prepared using ultrapure deionized water.

### Culture conditions for inoculum and strain maintenance

**Fig. 1:**
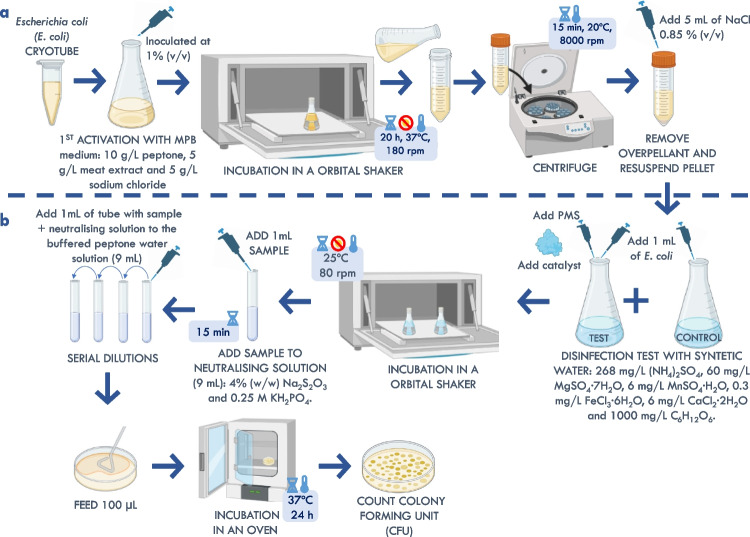
Outline of the experimental procedure for carrying out the disinfection assay. Section (**a**) depicts the activation and proliferation process of *E. coli*, while section (**b**) illustrates the procedure for conducting a batch flow disinfection assay (created in BioRender.com)

The procedure described in Fig. 1a was followed to activate the microorganism and ensure optimal inoculum conditions for performing the disinfection test, regarding viability and proliferation. First, 0.5 mL of the cryotube with the cryopreserved strain (inoculum at 1% (v/v)) and 50 mL of previously sterilized MPB medium (autoclave at 121 °C and 1.5 atm for 15 min; J.P. Selecta®, Presoclave II) were introduced into a 250-mL Erlenmeyer flask with a cellulose cap. Then, the Erlenmeyer flask was placed in the orbital shaker (Thermo Electron Corporation, Forma Orbital Shaker), and the flask was incubated for 20 h at 180 rpm, 37 ± 1 °C, and without light. After that time, the cells were in a stationary phase (12–24 h) with a minimum optical density of 1 at 600 nm (Blanco-Canella et al. 2022). Therefore, the entire contents of the flask were transferred to a sterilized Falcon tube, which was then centrifuged (Sigma Laboratory Centrifuges, 3K18) for 15 min at 8000 rpm and at ambient temperature (22 ± 3 °C). At the end of the centrifugation process, the supernatant was removed, to avoid any interference in the disinfection assays. Finally, the cell pellet was washed with 5 mL of saline solution, resulting in a 10-fold concentration. All assays were performed in aseptic conditions.

### Disinfection in a discontinuous system

Figure 1b shows the whole process for performing a disinfection assay. Once the inoculum was ready to be used (following previous sections), batch flow disinfection assays were conducted and the viable bacteria were quantified in colony-forming units (CFU). Therefore, 1 mL of the cell pellet (with a minimum optical density of 1 at 600 nm (around 10^10^ CFU/mL)) was added to a 250-mL Erlenmeyer flask that contained 99 mL of synthetic wastewater, after sterilized by vacuum filtration (0.22 μm) (Fdez-Sanromán et al. 2022a). Then, the amount of copper catalyst was added first, followed by the PMS solution, which were assessed during the test. Subsequently, the flask was homogenized (by performing 5 enveloping movements) and left to incubate without light in an orbital shaker (Thermo Scientific, MaxQ 8000) at 80 rpm, 25 ± 1 °C. All assays were performed in aseptic conditions.

To evaluate the disinfection process, a 1-mL sample was taken from the flask, previously homogenized, and added to a tube with neutralizing solution (9 mL of neutralizing solution; dilution 1:10^1^). The sample was left to act for 15 min with the added solution to halt the reaction (Raffellini et al. 2011). After this time, serial dilutions were performed on the sample in tubes containing 9 mL of buffered peptone water solution (serial dilution). To finish, 100 μL of each dilution was seeded by extension in a Petri dish of MPB-agar medium (with each sample in triplicate) and incubated in an oven (Memmert, Un 160) at 37 ± 1 °C for 24 h, for the subsequent recount of CFU.

In these assays, a control assay was always included, where the Erlenmeyer flask contained 99 mL of synthetic wastewater and 1 mL of the resuspended cell pellet. The control flask was incubated under the same aseptic conditions, and a sample was collected whenever the test flask was sampled. This was done to confirm that the microorganism was in perfect condition during the disinfection test.

It should also be noted that a sample (4–5 mL) was taken out in these batch flow disinfection tests to determine copper leaching by the catalysts.

### Synthesis of polymer composite beads

**Fig. 2:**
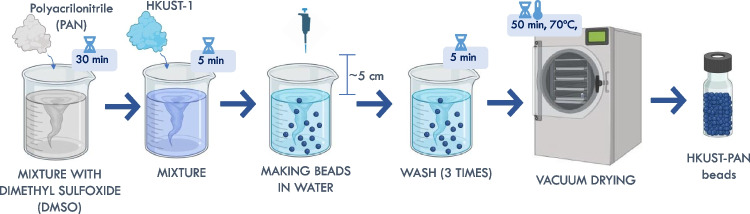
Schematic of the HKUST-PAN bead manufacturing process (created in Biorender.com)

To improve the handling of the HKUST-1 powder in flow systems, it was decided to encapsulate the HKUST-1 in PAN beads (HKUST-PAN) and compare it with the use of the powder phase. The synthesis process of the HKUST-PAN spherical beads, described in Fig. 2, was adapted from the reported procedure by Riley et al. (2020). In addition, the details of the synthesis ratios of HKUST-1 and PAN can be found in Table 2. It is worth mentioning that the ratio of PAN and DMSO corresponds to 1:15 (g PAN/mL of DMSO).

**Table 2:** Amounts of the reagents for composite preparation of the HKUST-PAN beads. ^*^Determined by ICP analysis

Used in the beads preparation				Final amount in beads*	
HKUST-1 (mg)	PAN (mg)	DMSO (mL)	HKUST-PAN ratio (%)	HKUST-1 (mg)	HKUST-PAN ratio (%)
22.5	200.9	3.0	10	16.95	8
134.4	200.9	3.0	40	84.92	30
488.2	200.7	3.0	70	301.98	60
1201.6	133.5	2.0	90	551.93	80

### Disinfection in a continuous system

**Fig. 3:**
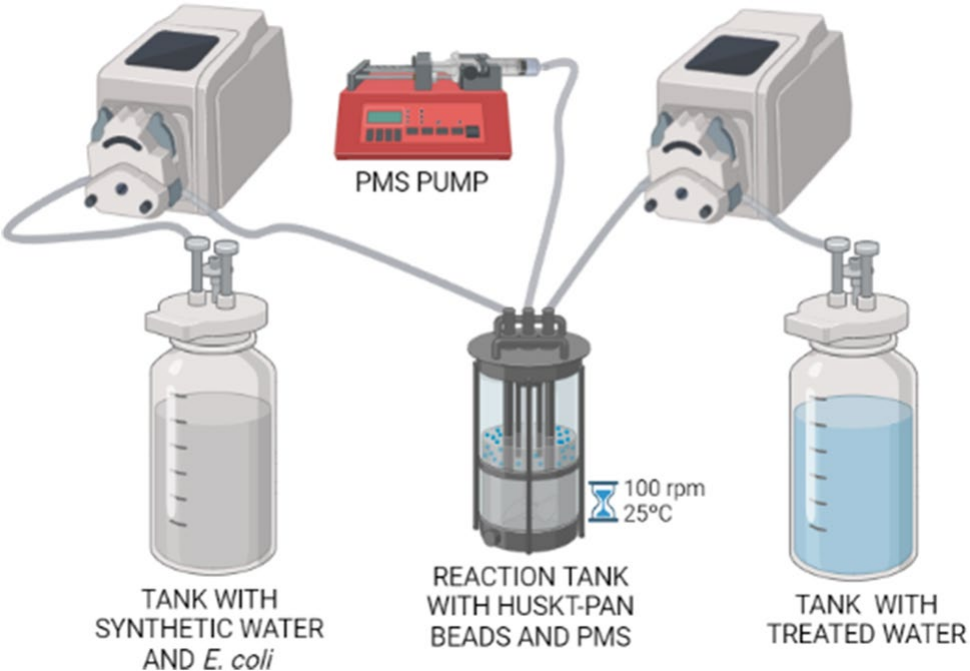
Diagram of the continuous flow system designed for the disinfection assays (created in Biorender.com)

To evaluate the scalability of the disinfection process, as well as the reusability of the immobilized catalyst, a reactor working in a continuous flow was designed (Fig. 3). In this system, an attempt was made to scale up the conditions previously obtained as optimal in the system with a batch flow (0.1 mM PMS and 60.5 mg/L of HKUST-1 immobilized in 30%HKUST-PAN beads).

As shown in Fig. 3, the continuous flow system was characterized by a 3.5-L inlet tank with a working volume of 2.5 L of synthetic water and *E. coli*. This tank was inoculated at 1% (v/v), following the inoculum proliferation described in the “Culture conditions for inoculum and strain maintenance” section. The reaction tank was a 250-mL stirred tank that operated with a working volume of 100 mL, composed of synthetic water, the inoculum (1% v/v), and PMS. In addition, the operating conditions of this reactor were 25 °C and 100 rpm, using mechanical agitation. The system also had an outlet tank with the same characteristics as the inlet tank.

Regarding the working mode of the system, it should be noted that the working volume and the immobilized catalyst were introduced into the reactor and allowed to reach the stationary state to start it up. The selected hydraulic retention time was 1 h, so the inlet and outlet flow rate of the peristaltic pumps (Masterflex L/S, Easy-load II 77200-50) was 1.67 mL/min and 33.4 μL/h of the PMS syringe pump (Aladdin SyringeONE Programmable Syringe Pump, Al-1000).

To evaluate the disinfection process, samples were taken every hour, removing one sample for the CFU count (1 mL, following the procedure described in the “Disinfection in a discontinuous system” section) and another for the determination of copper leaching (5 mL).

### Scavenging assays

To determine the mechanism of action of the HKUST-1 and PMS system and to identify which ROS were involved in the process, as well as the contribution made by each, a series of selective inhibition assays were performed. The assays were conducted using a model pollutant, RB5, because the scavengers used are toxic for the microorganisms.

These tests consist of applying a series of compounds that were able to annul some of the species present in the reaction (Wang and Wang 2020). In that way, the inhibitors did not change the overall outcome of the process, but locked a specific compound, the one whose activity was to be known. Therefore, any difference between the original process and the selective inhibition test could be assumed to be caused by the species that was inhibited.

There are multiple inhibitors capable of intercepting the produced ROS in PMS systems before they interact with the pollutants to degrade them (Wang et al. 2021). Therefore, selective inhibition experiments could be designed to discern the mechanism of action of this relatively complex system.

In this case, methanol (1 mM) was chosen as an inhibitor to determine free radical species (SO_4_^●−^/OH^−^), KI (1 mM) to determine the reactive surface complex, and NaN_3_ (1 mM) to determine singlet oxygen (^1^O_2_) (Guan et al. 2018; Wang et al. 2019).

To evaluate the action of these species, several degradation tests were carried out using a 50-mL beaker with a working volume of 25 mL. The volume was composed by the pollutant (25 ppm RB5), the oxidant (0.1 mM PMS), the inhibitory species to be evaluated in that test (1 mM), and the catalyst (60.5 mg/L HKUST-1).

In addition, to determine the catalytic capacity, a degradation control test was carried out with the same working volume composed of the pollutant, oxidant, and catalyst. Furthermore, to know the action of the oxidant, another degradation control test was carried out (PMS control), which was composed only of the pollutant and the oxidant.

All these tests were also carried out in duplicate in order to reduce measurement error.

### Analytical methods

#### Disinfection efficiency

For the assessment of the disinfection efficiency, the colonies were quantified by the standard plate count method (also known as CFU method) using a 10-fold serial dilution as described in the “Disinfection in a discontinuous system” section and Fig. 1b. The seeding method used for each sample dilution was the extension method, which was carried out in triplicate. After 24 h of incubation at 37 ± 1 °C, the CFU were counted manually. The detection limit between 20 and 300 CFU was set according to Blanco-Canella et al. (2022). Equation (4) was applied to determine the CFU/mL by multiplying the average value of colonies obtained on plates of the same dilution for a given sample by 10 to the power of the corresponding dilution number (*n*), multiplied by 100 (a 1:100 dilution was used) and divided by the volume of the seeded sample (0.1 mL). Additionally, the average colony values obtained were always averaged with a coefficient of variation for each sample dilution that was less than 20%.

4 $${~}^{{CFU}}\!\left/ \!{~}_{{mL}}\right.=\frac{\mathrm{CFU}\ \mathrm{average}\ \mathrm{dilution}\times {\mathrm{10}}^{\mathrm{n}}\times \mathrm{100}}{\mathrm{volume}\ \mathrm{seed}}$$

Finally, the logarithm of the CFU/mL values obtained was plotted in relation to the duration of the disinfection process.

#### Cell growth of the inoculum

*E. coli* concentration was determined by measuring the absorbance at a wavelength of 600 nm (Thermo Scientific^TM^, UV-Vis Genesys^TM^ 140/150). Previously, to be able to interpret the absorbance values, a calibration curve was made, in which the concentration of the dry weight of the cells was related to the optical density.

#### Evaluation of dye removal in the scavenging tests

The scavenging tests were monitored by taking aliquots to measure the sample absorbance. These samples were filtered (0.22-μm hydrophilic PTFE) before transferring them to the spectrophotometer cuvette, to remove any catalysts particles that could interfere with the absorbance value measured. These absorbance measurements were carried out at a wavelength of 596 nm (absorbance measurement of the highest peak height of RB5), thus obtaining the concentration of the dye present in the solution at a given time. The obtained elimination results were plotted by representing the percentage of dye removal against time, obtained using Eq. (5), where *C*_0_ was the initial dye concentration and *C* is the dye concentration of the sample at a specific time.

5 $$\mathrm{Dye}\ \mathrm{removal}\ \left(\%\right)=\left(1-\frac{C}{C_0}\right)\times 100\kern0.5em$$

As a preliminary step, a calibration curve was made relating different dye concentrations and the maximum absorbance at 596 nm.

#### Microscopy analysis

To determine the morphology and composition of the HKUST-1 and HKUST-PAN beads, before and after the tests, microstructure analyses were conducted. To do this, a scanning electron microscope (SEM) was used, where the samples were analyzed with a gold coating (approx. 15 nm). On the other hand, for the energy-dispersive X-ray spectroscopy analysis (EDS), the samples were coated with carbon (approximately 10 nm). In addition, some of the HKUST-PAN beads were cross-sectioned for the analysis of their interior.

All these analyses were carried out at the *Centro de Apoio Científico-Tecnolóxico á Investigación* (C.A.C.T.I.) of the University of Vigo, using JEOL JSM6010 and FEI-Quanta 200 equipment, applying an accelerating voltage of 10–15 kV (Ferreira et al. 2013; Fdez-Sanromán et al. 2023).

#### Copper analysis

To ascertain of copper content in the HKUST-PAN beads and its release in the medium following disinfection testing, an inductively coupled plasma optical emission spectrometry (ICP-OES) analysis was performed. The ICP-OES iCAP PRO equipment located at the C.A.C.T.I. was utilized for this analysis. The instrument has a detention limit of 0.003 mg/L. In the case of HKUST-PAN beads, before measurement, acid digestion was performed, following the procedure described by Pazos et al. (2010).

#### X-ray diffraction analysis

For the crystallographic analysis of HKUST-1, X-ray diffraction spectrometry (XRD) was performed at the C.A.C.T.I. facility using the XPert Pro diffractometer (PANalytical) with Cu Kα radiation (λ = 1.54 Å). The data were collected using the Bragg-Brentano geometry in the range theta-2theta (θ) = 5–70° with a step time of 120 s and a step size of 0.026° and using a current of 30 mA and a voltage of 40 kV in the X-ray tube.

#### Thermogravimetric analysis

To further characterize HKUST-1, a thermogravimetric analysis (TGA) was performed using the TGA-DSC SETSYS Evolution 1750 (Setaram) at the C.A.C.T.I. The sample to be analyzed was heated from 20 to 800 °C, at a heating rate of 10 °C/min, and the measurements were carried out in an inert atmosphere (N_2_).

## Results and discussion

### Preliminary disinfection assays

**Fig. 4:**
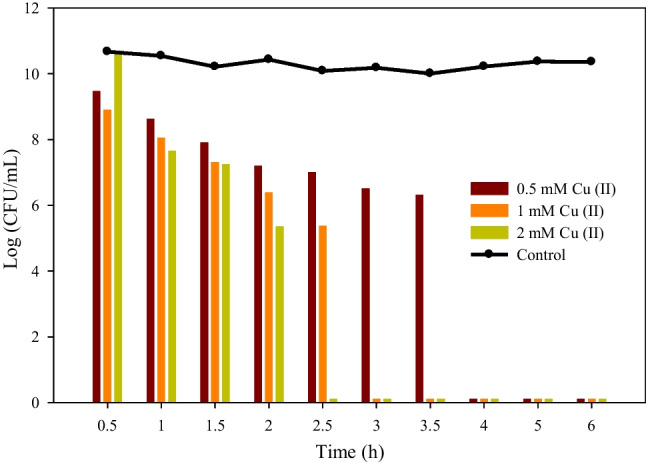
Screening results of homogenous copper disinfection tests (0.5, 1, and 2 mM Cu (II)). The line represents the control results

Initially, several control assays were carried out to evaluate the disinfecting potential of HKUST-1 against *E. coli*. A Cu (II) solution was initially used in the control tests to evaluate its antibacterial activity as it is traditionally used to control microbiological contamination at high concentrations (Chiericatti et al. 2012; Cun et al. 2022). Therefore, different concentrations of Cu (II) (0.05, 0.25, 0.5, 1, and 2 mM) were tested in these assays to evaluate their efficacy. At low Cu (II) concentrations (0.05 and 0.25 mM), *E. coli* growth persisted even after 24 h of culture. In contrast, Fig. 4 shows that when Cu (II) concentrations were higher (0.5, 1, and 2 mM) in the assays, *E. coli* growth was entirely suppressed after 4, 3, and 2.5 h, respectively. Moreover, the obtained growth curves exhibited a conspicuous decrement in the logarithmic profile. According to Salah et al. (2021), the effect of the copper ions seems to be multifaceted, with the ROS generation being the main mechanism of bactericidal activity that irreversibly damages membranes.

Subsequently, a test was carried out to evaluate the *E. coli* disinfection potential of heterogeneous copper, in this case using the HKUST-1. To provide context, a concentration of 2 mM Cu (II) was used to determine the corresponding concentration in HKUST-1 (403.2 mg/L). As the disinfection process took the shortest time possible, the results allow comparison between the two tests.

**Fig. 5:**
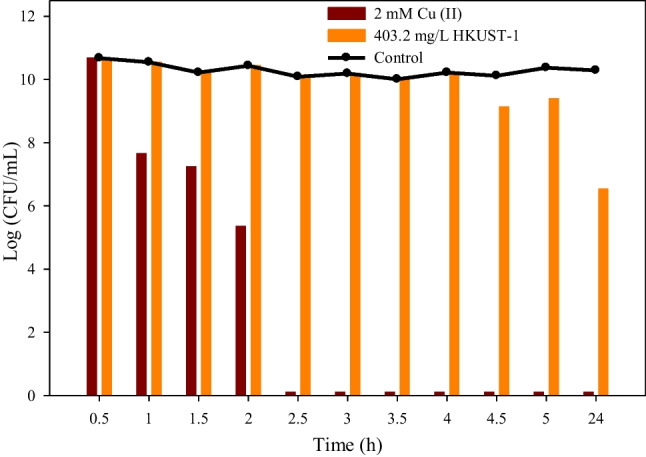
Screening comparison of the disinfection test with 2 mM Cu (II) and 403.2 mg/L HKUST-1

Based on the results of Fig. 5, cell growth did not decrease with this concentration of HKUST-1 until about 4.5 h after the exposure, and the bacteria were not eliminated after about 24 h. This finding aligns with Chiericatti et al.’s (2012) report on the use of this biocidal substance against typical yeast and mold. Thus, a higher concentration of HKUST-1 is needed to eliminate cell growth. Furthermore, these tests verify the bactericidal power of HKUST-1 and establish, similar to copper in the homogenous phase, that complete elimination of microorganisms requires a high concentration.

### Disinfection using PMS and HKUST-1

**Fig. 6:**
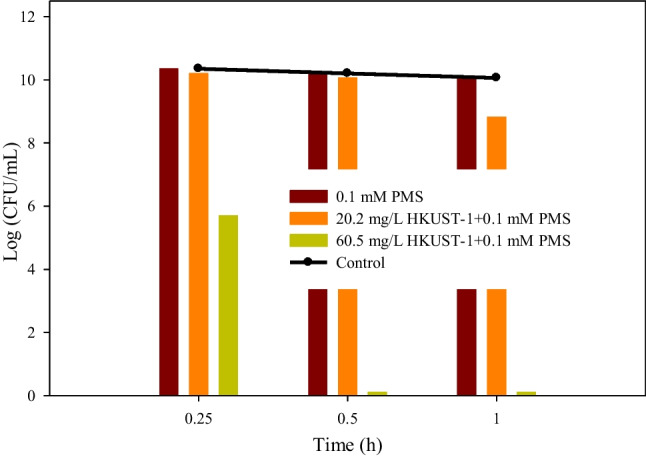
Evaluation of disinfection tests with PMS (0.1 mM) and the same concentration activated with HKUST-1 (20.2 and 60.5 mg/L)

After evaluating the disinfection potential of the biocidal material, its catalytic activity for generating sulphate radicals was evaluated. Thus, new tests were performed to assess the disinfection ability of the combination of PMS and HKUST-1. The results were compared to those of PMS with free Cu (II). In these tests, the concentrations of the catalysts previously determined as optimal (2 mM Cu (II) and 403.2 mg/L HKUST-1) and 2 mM PMS (a molar ratio of 1:1 with respect to free copper) were used. Both assays resulted in the complete elimination of cell growth after 30 min of testing. While these results established the catalytic action of copper and higher bactericidal activity of activated PMS compared to previous studies, it must be noted that high concentrations of PMS alone could be sufficient to eliminate cell growth. Therefore, control tests were conducted using only PMS to verify the disinfectant capability of the oxidant. As demonstrated by Fdez-Sanromán et al. (2022b), cell growth was eliminated after 30 min with 0.5 and 0.3 mM PMS. Hence, it was decided to perform a test using a concentration of 0.1 mM PMS. After performing these tests, it was observed that more than 1 h was required for the complete elimination of cell growth during the disinfection process (Fig. 6). Therefore, it was necessary to optimize the concentration ratio of PMS and catalyst to be used.

Owing to the fact that both catalysts, homogeneous and heterogeneous, obtained positive results, it was decided to optimize only the concentration of the heterogeneous catalyst HKUST-1. This was because HKUST-1 is an alternative, novel catalyst and presents greater possibilities in the real scaling-up of the process. Therefore, new assays were designed to optimize the disinfection process using the fixed PMS concentration of 0.1 mM and a variable HKUST-1 concentration (20.2 and 60.5 mg/L). The obtained results can be seen in Fig. 6, where a pronounced decreasing logarithmic profile of the cell growth can be seen in the assays with a concentration of 60.5 mg/L HKUST-1 and 0.1 mM PMS. Concerning the control assay of the microorganism, in all the assays, it was obtained with practically equal growth.

Furthermore, it should be noted that the assay with 0.1 mM PMS and 60.5 mg/L HKUST-1 was considered the best condition, because after 15 min, a significant biocidal activity was detected, and cell growth was eliminated after 30 min.

In Table 3, the significance of our study was denoted by comparison to existing studies. Thus, the proposed system’s high performance with regard to treatment time and reagent concentration (catalyst and oxidant), compared to recent studies on *E. coli* disinfection using sulphate radicals, was demonstrated.

**Table 3:** Comparison of the disinfection of *E. coli* by different heterogeneous catalysts used in sulphate radical activation in relation to this study

Catalyst	Time (min)	Catalyst (mg/L)	Oxidant (mM)	Disinfection efficiency (%)	References
HKUST-1	30	60.5	0.1 PMS	100	This research
CuFe-(BDC-NH_2_)_R_	60	250	0.1 PMS	100	Fdez-Sanromán et al. (2023)
FeCl_3_-biochar	20	500	0.11 PS	>99	Zeng and Kan (2023)
g-C_3_N_4_	120	100	0.05 PS	100	Zhang et al. (2021)
CuFe_2_O_4_	180	800	4 SP	100	Qin et al. (2023)
Tenorite (CuO)	15	1000	0.1 PDS	100	Li et al. (2022)
Ilmenite (Fe_2_Ti_4_O_3_)^×^	20	1000	0.05 PS	100	Xia et al. (2018)

^×^Combined with UV

*PS*, persulphate; *PDS*, peroxydisulphate; *SP*, sulphite

### Characterization HKUST-1 powder

**Fig. 7:**
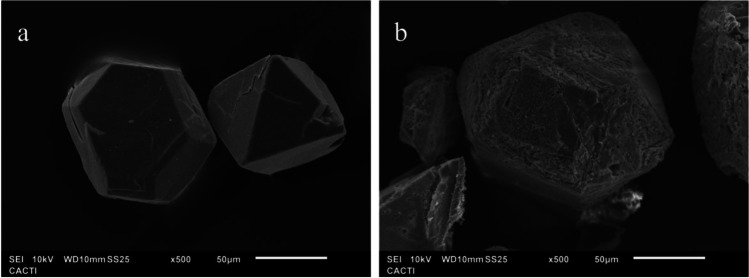
Microstructures of the samples: initial sample of HKUST-1 (**a**) and test sample 20.2 mg/L HKUST-1 + 0.1 mM PMS (**b**)

The characterization of the catalyst before and after the treatment was performed in order to evaluate its stability. The results of the SEM microscopy test (Fig. 7) showed changes in the morphology of HKUST-1 after being used in the different disinfection tests, but not in the size of the crystal. Figure 7a shows the typical uniform octahedral shapes, micrometer-sized, with a smooth surface and sharp edges (Chiericatti et al. 2012) which were modified after the treatment using PMS (Fig. 7b). In addition, craters can be observed on the eroded surface of the crystals after treatment, in agreement with several reports (O’Neill et al. 2010; Harvey et al. 2011; Kolay et al. 2023).

In addition, the supplier provided HKUST-1 with a specific surface area (BET) of 1500–2100 m^2^/g and with a bulk density of 0.35 g/cm^3^, which agrees with the reported values in the literature (Najafi Nobar 2018; Guo et al. 2021).

**Fig. 8:**
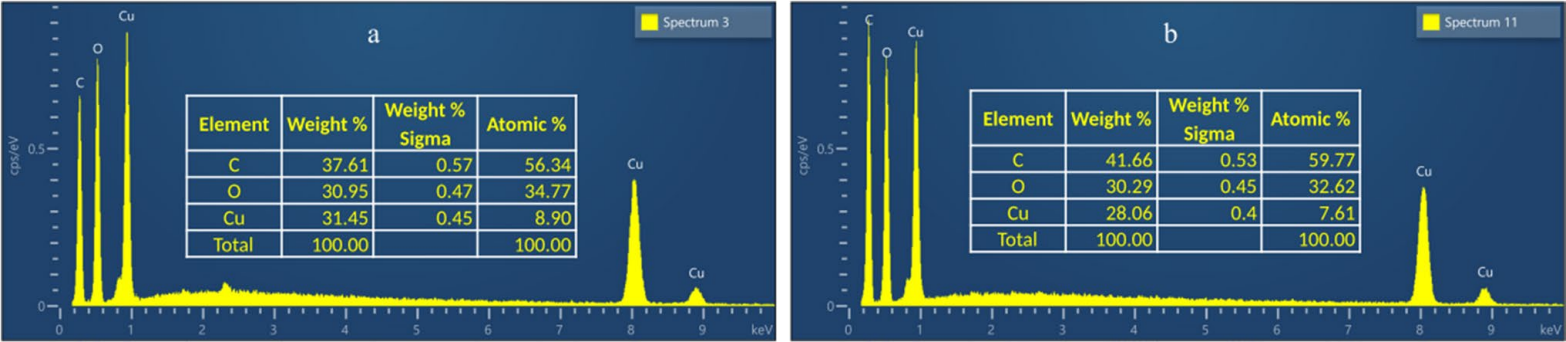
Based on the submitted samples, EDS spectra of the initial sample of the HKUST-1 (**a**) and HKUST-1 after the test with 0.1 mM PMS and 20.2 mg/L HKUST-1 (**b**)

On the other hand, the EDS studies (Fig. 8) showed a decrease in copper content in the samples before and after disinfection. The percentage of copper present in HKUST-1 was 31% in the unused sample and decreased to around 28% after use. The leaching of copper was evaluated by ICP-OES during the assays. High copper leaching (7.08 mg/L) was obtained after the disinfection tests using PMS activated with HKUST-1 in powder form. This value is comparable to the control test using only HKUST-1 and to the data reported by Chiericatti et al. (2012) in the literature. Therefore, it was postulated that the used PMS concentration did not provide high damage to the HKUST-1 structure. Thus, taking into account the copper leaching and the modifications of the HKUST-1 structure observed, it confirmed what Wyszogrodzka et al. (2016) reported about the bactericidal mode of action of HKUST-1. Therefore, this nanostructure material can also act as a reservoir of metal ions, providing their gradual release and resulting in sustained antibacterial action.

**Fig. 9:**
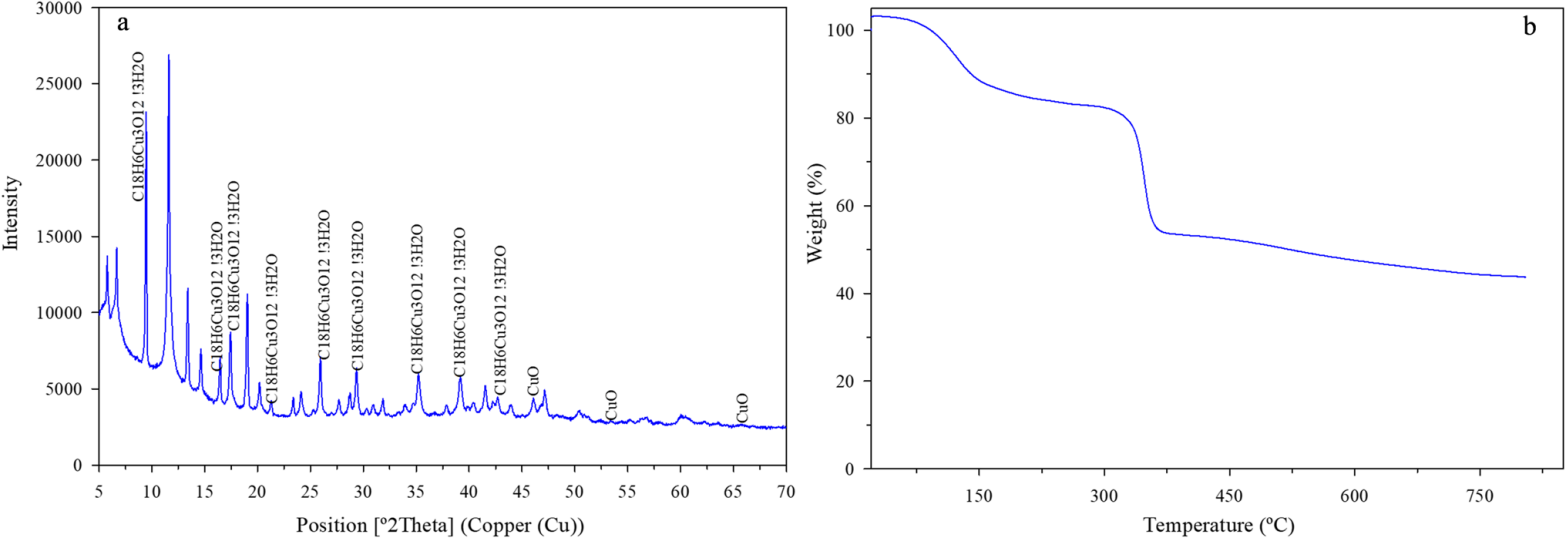
Results of the C.A.C.T.I. characterization of the MOF after the analyses of XRD pattern of the HKUST-1 (**a**) and TGA analyses of HKUST-1 (**b**)

Additionally, XRD analysis was performed to verify the high purity of the crystalline phases of the HKUST-1. The intense peaks shown in Fig. 9a appearing at small 2θ angles are characteristic of microporous materials, which possess numerous tiny pores or cavities, and are consistent with published data (Schlichte et al. 2004; Panella et al. 2006).

The thermogravimetric curve obtained was consistent, with significant weight loss steps as shown in Fig. 9b and agrees with what was reported in the literature (Lin et al. 2012). In the first part of that figure (until 150 °C), it can be seen how a weight loss of about 20% occurred, due to evaporation of water or host molecules. The molecular formula of the HKUST-1 was assumed to be Cu_3_(BTC)_2_(H_2_O)·!H_2_O, where the number of crystals (!) is approximately 3. Afterwards, the sample remained practically stable up to about 300 °C, thus confirming the stability of the structure at higher temperatures and the low presence of impurities in its structure. From 300 °C onwards, a sudden weight change (around 30 wt.%) was observed up to 450 °C, due to the total damage of the BTC linker. Finally, it gradually lost about 10 wt.% up to 650 °C.

### Scavenging assays

To evaluate the radical species involved in the disinfection process, scavenging assays were performed. The concentrations used in these tests for the oxidant (PMS) and the catalyst (HKUST-1) were those obtained as optimal in the disinfection tests (0.1 mM PMS and 60.6 mg/L HKUST-1). For these assays, the model dye RB5 was used, because the scavenging compounds were toxics for the microorganisms. The scavenging tests consist of performing a degradation test with each one of the inhibitory species in the presence of HKUST-1 and PMS assay.

**Fig. 10:**
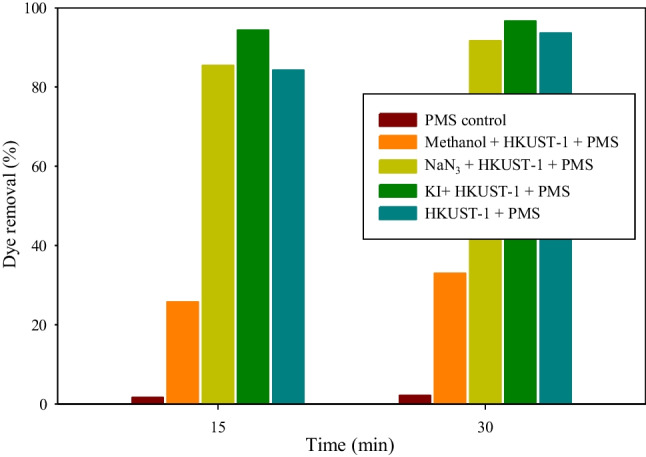
Evaluation of the scavenging tests by the effect of RB5 removal

To be able to evaluate the improvement when using a catalyst for the degradation of a pollutant, a test with only RB5 and PMS, called PMS control test, was carried out. As can be seen in Fig. 10, no significant removal was achieved after 30 min. In addition, observing Fig. 10, it can be seen that when the action of the free radicals (SO_4_^●−^/OH^−^) is blocked (in the presence of methanol), the dye removal reaches its maximum after 30 min (dye removal of approximately 32%) and the reaction is completely stopped. On the opposite, when singlet oxygen (in the presence of NaN_3_) or surface reactive compounds (in the presence of KI) were inhibited, the reaction stopped at 15 min. These results exhibited minimal variance compared to the standard degradation test (HKUST-1+PMS) when the NaN_3_ was utilized. However, for KI, some differences were obtained in the degradation assays associated with the adsorption assay. Therefore, it was concluded that the main activation mechanism of the HKUST-1 and PMS system is radical action.

Based on all previous findings, the encapsulation of HKUST-1 in beads made of PAN fibers was assessed to simplify handling and enable potential reuse, considering that low particle size can hinder the reusability of this nanostructure material as suggested by prior research.

### Disinfection using PMS and HKUST-PAN beads

**Fig. 11:**
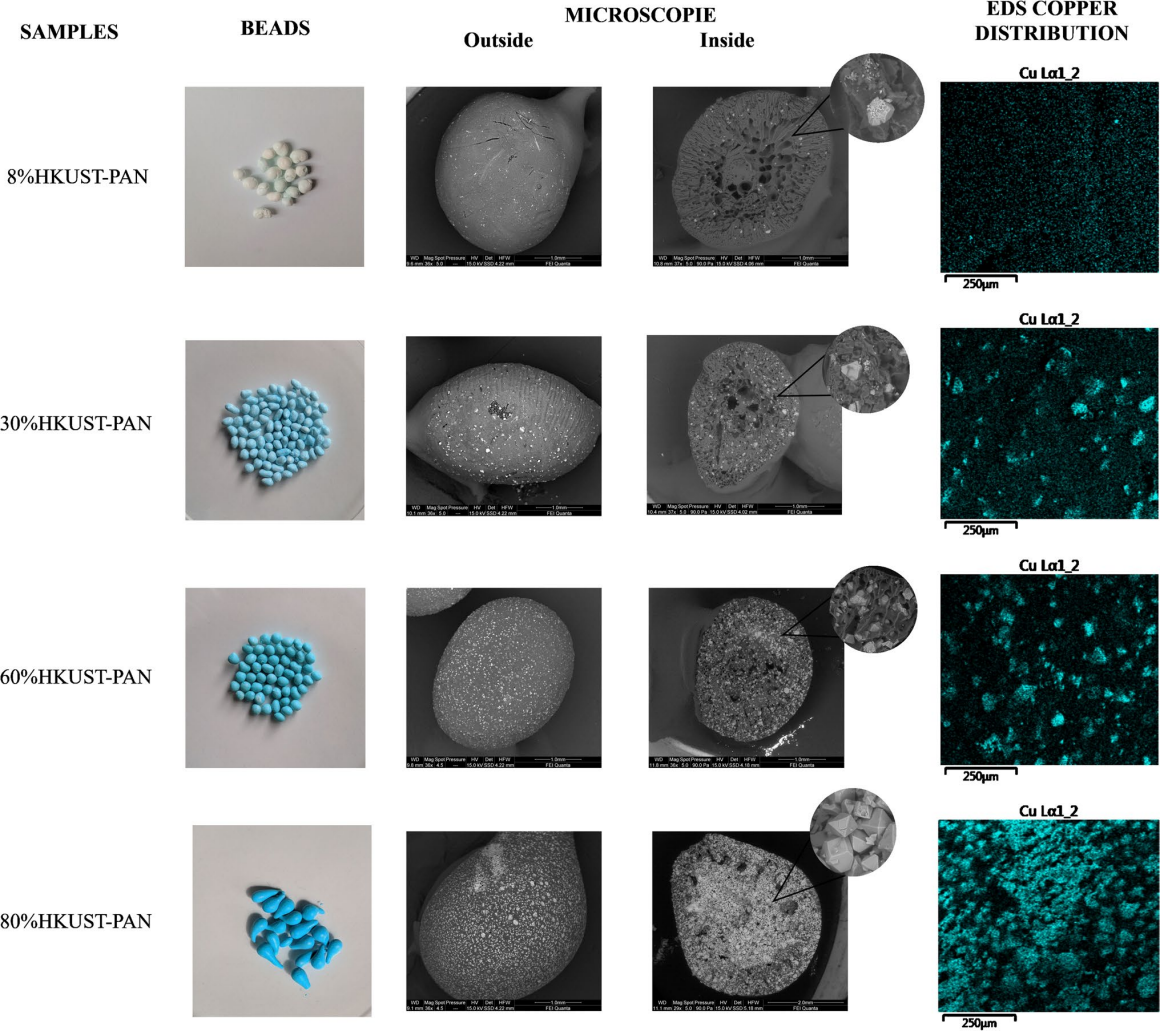
Photos, SEM images, and details of microstructures and EDS copper distribution of HKUST-PAN beads synthesized with different HKUST-1 concentrations

SEM-EDS was used to perform a detailed physical and chemical characterization of the different synthesized beads. The appearances of the HKUST-PAN beads are shown in Fig. 11. As the HKUST-1 loadings increased, the color of the beads became deeper shades of blue, according to Riley et al. (2020). The size distribution of the final beads depends on the MOF concentration. Typically, beads with higher concentrations show smaller sizes; however, the size distribution ranged from 0.4 to 0.3 cm. The cross-section images acquired from SEM reveal some details of the synthesized composites and the distribution of HKUST-1 crystals. Moreover, the cross-sectional views of whole bead were polyhedral and generally vary from ∼50 to 100 μm in size. The distribution of MOF was even throughout the beads, revealing the PAN pore network and embedded HKUST-1 crystals within the composites. The interior of 60% and 80%HKUST-PAN beads was tightly packed with crystals.

Additionally, it was possible to observe, in the microstructures of the beads in Fig. 11, the difference in porosity between the outer surface of the bead and its interior, being able to appreciate mesopores, micropores, and reticular channels in the cross-section of these beads. Besides, in the detail of the cross-section, it could also be observed that this porosity increased with the increase of HKUST-1 concentration present in the bead, agreeing with what was reported in the literature (Riley et al. 2020).

As is shown in Fig. 11, EDS mappings display a uniform distribution of the HKUST-1 in the PAN beads for all synthesized materials. 8%HKUST-PAN beads presented a homogenous distribution, while the other catalysts showed some HKUST-1 loaded areas in accordance with the concentration of MOF. The crystals perfectly adhered to the polyacrylic fibers. Hence, it can be stated that the PAN matrix acts as a firm net that stabilizes the crystals in place. Similar observations were reported in previous studies (Riley et al. 2020).

**Fig. 12:**
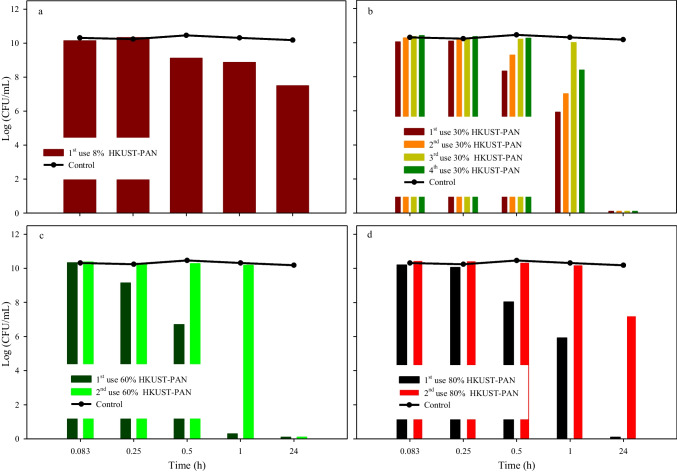
Evaluation of disinfection tests using a concentration of 60.5 mg/L HKUST-PAN beads and 0.1 mM PMS, evaluating the catalytic action and their possible reuse in these tests, of the different concentrations of HKUST-1 used in the manufacture of the beads: **a** 8%HKUST-PAN, **b** 30%HKUST-PAN, **c** 60%HKUST-PAN, and **d** 80%HKUST-PAN. Note that 1st, 2nd, 3rd, and 4th mean the number of uses of HKUST-PAN beads in that disinfection test

**Fig. 13:**
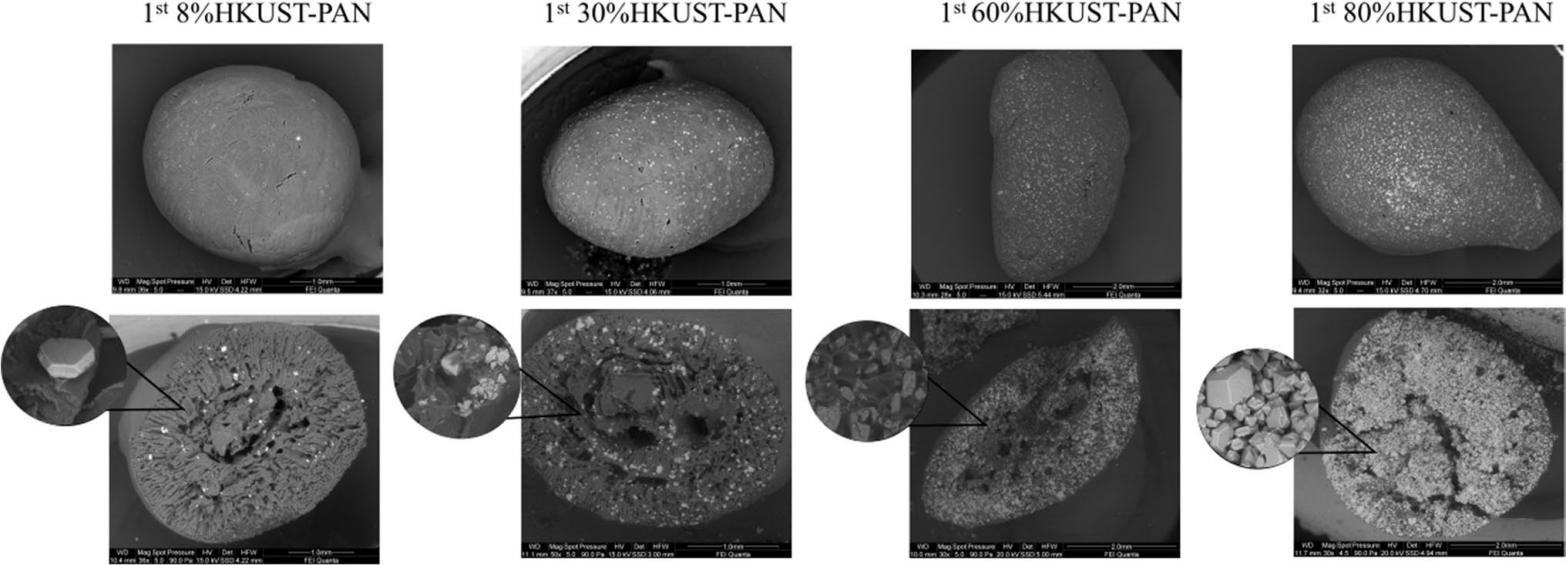
SEM images and detail of microstructures of HKUST-PAN beads after the first reuse

After characterization, the catalytic activity of the synthesized HKUST-PAN beads was examined at the previously determined optimal concentration of 60.5 mg/L HKUST-1 and 0.1 mM PMS. These assays were deemed satisfactory since a decline in cell growth was observed after 15 min, and after 24 h, complete elimination of cells was observed, as shown in Fig. 12. However, this was not the case when using the beads with a very low concentration of HKUST-1 (8%HKUST-PAN beads) (Fig. 12a). In addition, as shown in Fig. 13, all beads exhibited excellent physical stability after their initial use. Therefore, the beads were evaluated for reuse in a second cycle under the same conditions, after a reconditioning process (2 washes in 100 mL distilled water with vigorous shaking for 5 min each and a subsequent drying at 70 °C for 2 h). In this second use of the beads, it was observed that assays utilizing 80%HKUST-PAN beads failed to eliminate cell growth after 24 h (Fig. 12d). It was postulated that an excess of catalyst could have a detrimental effect on the action of PMS. Regarding the tests using 60%HKUST-PAN beads (as depicted in Fig. 12c), cell growth was eliminated after 24 h. However, higher disinfection rates were observed when using 30%HKUST-PAN beads (Fig. 12b).

For this reason, it was decided to evaluate what happened with a third and fourth use of 30%HKUST-PAN beads. Based on previous results, it was determined that the growth decay time increased after disinfection with new beads compared to used beads. Nonetheless, growth was eliminated after 24 h using both cases. Moreover, copper leaching by ICP analysis was less than 5%.

**Fig. 14:**
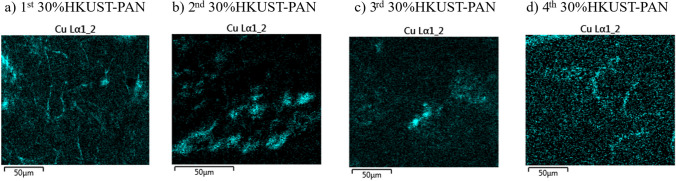
EDS copper distributions in each of the different 30%HKUST-PAN beads after different cycles

After several uses, the 30%HKUST-PAN beads were thoroughly analyzed by SEM and EDS cooper distribution was evaluated (Fig. 14). Analysis of the cross-section of the 30%HKUST-PAN beads revealed the presence of HKUST-1 crystals, exhibiting the same morphology and distribution as mentioned earlier. Furthermore, EDS mappings demonstrate that copper was present in the beads and the homogenous distribution of copper content (4–3%) remained unchanged without any significant variations. This demonstrates the viability of the developed catalyst for use in continuous mode in disinfection treatments.

To confirm this hypothesis, an experimental set-up of a continuous reactor, as it was described in the “Disinfection in a continuous system” section and Fig. 3, was accomplished using as catalyst 30%HKUST-PAN beads.

**Fig. 15:**
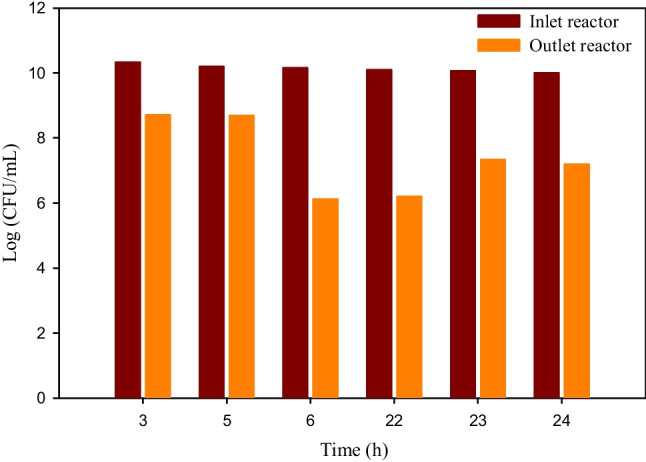
Evaluation of the disinfection test in a continuous flow system with 30%HKUST-PAN beads and PMS by CFU analysis in the reactor inlet and outlet flows

PMS addition was continuously performed by a syringe pump (33.4 μL/h) and the polluted water flow in the reactor at 1.67 mL/min, obtaining a hydraulic retention time (τ) of 1 h. In Fig. 15, it can be observed that, after reaching the stationary state (around 3 times τ), the system had a behavior that fits with the results obtained at 1 h in the batch system (Fig. 12b), and these results were stable over time. Therefore, if longer residence times are used, complete removal of the microorganism will be achieved. Moreover, the reusability of the catalyst was also verified, concluding that the results obtained in the batch flow test were maintained (4 cycles) and that, even after 24 h (24 cycles), the catalytic properties were still maintained.

Consequently, the reusability and scalability of the proposed system of treatment were demonstrated. To the authors’ knowledge, this is the first time that a continuous system for sulphate radical generation using MOF as a catalyst was developed which highlights the significance and novelty of the performed research.

## Conclusions

In this research, a deep study has been carried out to evaluate the performance of the disinfection using HKUST-1, in synthetic wastewater with high microbiological load, *E. coli.* For this purpose, microbial disinfection efficacy of the nanostructure copper-organic framework, as well as its action as catalyst of PMS in the generation of sulphate radicals, has been evaluated. According to the disinfection tests, it is concluded that the nanostructure HKUST-1 has been characterized as having a dual functional action, due to its high disinfectant power and catalytic action in the presence of PMS. In addition, it has been found that when the HKUST-1 was encapsulated in beads with PAN fibers, the disinfection results and the catalytic action of the MOF were maintained in different cycles achieving the best results with the composite 30%HKUST-PAN. Moreover, in this way, the copper losses obtained in the tests with HKUST-1 in the powder phase have been reduced to a great extent. Finally, it should be noted that this study may represent a breakthrough, since a robust heterogeneous catalyst system has been obtained for the elimination of pathogens using sulphate radicals, achieving a quick elimination of these and the possible reuse of the catalyst in multiple treatment cycles, as well as the possibility of scaling up the process to a continuous flow system.

## Data Availability

Data available on request from the authors.
